# Near-infrared and metal-free tetra(butylamino)phthalocyanine nanoparticles for dual modal cancer phototherapy†

**DOI:** 10.1039/d0ra03898a

**Published:** 2020-07-09

**Authors:** Ying-Jie Wu, Fan-Hong Lv, Jing-Lan Kan, Qun Guan, Anqi Xue, Quanbo Wang, Yan-An Li, Yu-Bin Dong

**Affiliations:** College of Chemistry, Chemical Engineering and Materials Science, Collaborative Innovation Center of Functionalized Probes for Chemical Imaging in Universities of Shandong, Key Laboratory of Molecular and Nano Probes, Ministry of Education, Shandong Normal University Jinan 250014 P. R. China kanjinglan@163.com yubindong@sdnu.edu.cn; Shandong Analysis and Test Center, Qilu University of Technology (Shandong Academy of Sciences) Jinan 250014 P. R. China

## Abstract

Synergistic phototherapy combining photodynamic therapy (PDT) and photothermal therapy (PTT) based on near-infrared (NIR) dyes using a single light source offers the opportunity to treat diseases at deep locations. In this study, we reported human serum albumin (HSA)-involving tetra(butylamino)phthalocyanine (Pc)-based nanomaterials of HSA-α-Pc and HSA-β-Pc as highly efficient dual-phototherapy agents, namely 1(4),8(11),15(18),22(25)-tetra(butylamino)phthalocyanine (α-Pc) and 2(3),9(10),16(17),23(24)-tetra(butylamino)phthalocyanine (β-Pc). Both HSA-α-Pc and HSA-β-Pc showed excellent photothermal effects under a single NIR (808 nm) laser irradiation due to the *S*_1_ fluorescence emission quenching of Pcs. Compared to HSA-β-Pc, HSA-α-Pc exhibited better singlet oxygen generation ability and its highly efficient PDT/PTT dual-phototherapy was also well evidenced *via in vitro* and *vivo* experiments under a single 808 nm laser irradiation. Overall, this approach would be viable for the fabrication of more new Pc-based metal-free nano agents for PDT/PTT synergistic phototherapy upon a single NIR light source.

## Abstract 2

Two new HSA-involved tetra(butylamino)phthalocyanine composite nanoparticles, as highly efficient dual-phototherapy agents upon a single NIR laser irradiation, were reported.

## Graphical abstract

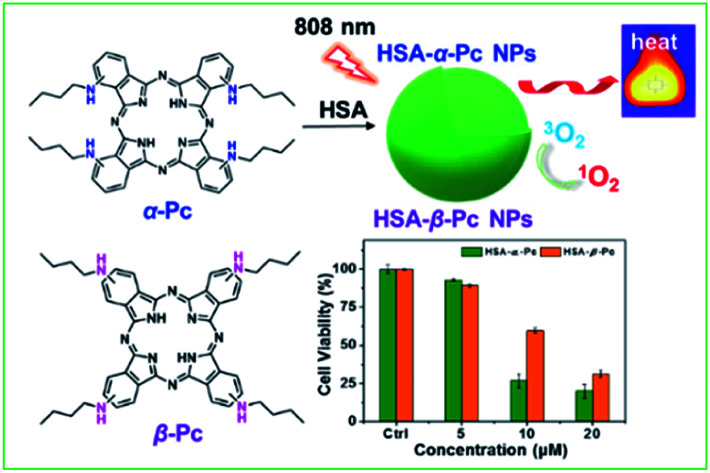

## Introduction

Phototherapy, including photodynamic therapy (PDT) and photothermal therapy (PTT), is a widely recognized approach for cancer treatment. PDT and PTT require light absorption and photosensitizers to generate reactive oxygen species (ROS) and heat to kill cancer cells, respectively.^1^ Different from PDT, PTT, as an oxygen independent phototherapy approach, can not only kill all the cancerous cells including hypoxic ones, but also increase the intratumoral blood flow and enriches tumor oxygenation for boosting the oxygen-elevated PDT.^2^ Therefore, the synergistic use of PDT and PTT dual-phototherapy can achieve optimized efficacy in treating almost all the malignant solid tumors. So far, the most reported PDT/PTT nanomaterials were constructed by combing typical organic photosensitizers (*e.g.* porphyrin, phthalocyanine, BODIPY, *etc*) with inorganic photothermal conversion agents (*e.g.* nano Au, carbon dots, metal sulfide, *etc*) through surface modification or encapsulation with multistep fabrication, low reagent loadings, and dual laser irradiation.^3^ So, the facile fabrication of single laser triggered, especially single near-infrared (NIR) laser induced PDT/PTT nano agents is very significant and imperative.

Phthalocyanines (Pcs) have been considered as a promising class of PDT materials because of their suitable *Q*_max_ band absorption (650–700 nm), high extinction coefficients, tunable photophysical and photochemical properties *via* facile chemical modifications.^4,5^ For example, aluminium Pc (Photosens®, Russia) has been approved for clinical use.^6^ In contrast, only a handful of Pc-based PTT examples have been reported so far. As it is known, the light-to-heat conversion efficiency of the Pc-based photothermal agents could be improved by effectively reducing or quenching their fluorescence and intersystem crossing by formation of the self-assembled Pc aggregation,^7^ synthesis of the paramagnetic metal (*e.g.* Cu^2+^, Fe^3+^, Co^2+^, Ni^2+^, *etc*) involved Pc metal complexes, and tuning the peripheral substituents (*e.g.* amino group through phenoxy as a bridge) or axial ligands (*e.g.* amino group through phenyl as a bridge) of Pcs.^8^ In some cases, Pcs not only serve as the ROS generation agent but also as the light-to-heat conversion agent by self-quenching excited states arising from the compactly packed monomer, which might allow the synchronous PDT/PTT under a single laser to be possible. For example, the hydrophobic Pc-loaded silica NPs (Pc@HSNs) reported by Li and co-workers exhibited efficient dual PDT and PTT effects,^9^ and Kim and co-workers recently reported the nanostructured Pc-assemblies (PcTBs) which displayed intrinsically unique photothermal and photoacoustic properties, but they all induced by the non-NIR Illumination (*λ* < 750 nm).^10^

An ideal agent for PTT/PDT combinatorial therapy should possess high photothermal conversion efficiency, meanwhile exhibit strong NIR (*λ* > 750 nm) absorption, which is a transparency window for biological tissues.^11,12^ However, the *Q*_max_ bands of the most Pc-based photothermal agents are located within 650–700 nm. To date, only one example of Pc-nanomaterial for NIR irradiated synchronous PDT/PTT was reported, in which the metal-involved ZnPc nanowire (NW) was prepared by a vaporization–condensation–recrystallization (VCR) multistep synthesis at 550 °C in Ar stream.^13^

**Scheme 1:**
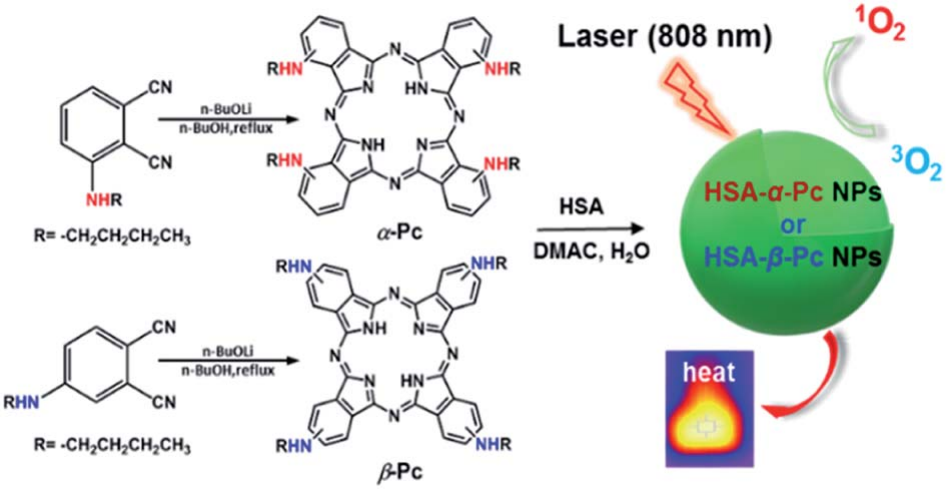
Schematic illustration of synthesis of HSA-α-Pc and HSA-β-Pc NPs and their phototherapy effects.

Our interest is to develop a facile approach for the fabrication of the metal-free Pc-based nano agents for dual modal cancer phototherapy upon single NIR laser irradiation. Besides NIR, the metal-free Pc-nanomaterials could completely avoid the toxicity potentially caused by metal species. In this contribution, two metal-free and butylamino group peripherally decorated NIR Pcs, namely 1(4),8(11),15(18),22(25)-tetra(butylamino)phthalocyanine (α-Pc) and 2(3),9(10),16(17),23(24)-tetra(butylamino)phthalocyanine (β-Pc), were reported. More importantly, their human serum albumin (HSA)^14^ assisted nanostructured assemblies of HSA-α-Pc and HSA-β-Pc were fabricated *via* a facile approach, and they all showed excellent photothermal effects under a single NIR (808 nm) laser irradiation. As HSA is the most abundant protein in plasma, it can serve as a versatile carrier for drug delivery. Currently, a number of clinical relevant HSA-based therapeutics have been approved by the Food and Drug Administration (FDA), and many more are under active clinical investigation.^15^ When being used to fabricate nano agents, it is unlikely to cause any undesired interaction with other serum proteins for these HSA-based nanomaterials. Thus, these nanomaterials with extremely low systemic toxicity have been studied as a versatile platform for diagnosis and precision therapy.^15–17^ For example, Abraxane fabricated *via* hydrophobic interactions between HSA and paclitaxel, a paclitaxel albumin nanoparticle, has been approved by FDA for treating various cancers.^18^ Of note, HSA-α-Pc displayed a better ROS generation ability than that of HSA-β-Pc, and its highly efficient PDT/PTT dual-phototherapy was well evidenced *via in vitro* and *vivo* experiments under a single 808 nm laser irradiation (Scheme 1).

## Experimental

### Materials

DMF was distilled from CaH_2_. All other commercial solvents and reagents were used without further purification unless otherwise mentioned. 3/4-Nitrophthalonitrile, *n*-butylamine, 1,3-diphenylisobenzofuran (DPBF), Li, and extra dry *n*-butanol were purchased from Energy Chemical Co., Ltd. Trimethylamine was purchased from Sinopharm Chemical Reagent Co., Ltd. Human serum albumin (HSA) and 3-(4,5-dimethyl-2-thiazolyl)-2,5-diphenyl-2*H*-tetrazolium bromide (MTT) were purchased from Sigma-Aldrich (Shanghai) Trading Co. Ltd. Phosphate-Buffered Saline (PBS) and Dulbecco's Phosphate-Buffered Saline (DPBS) were purchased from Biological Industries USA, Inc. RPMI Medium 1640 basic (1X) (RPMI 1640), Fetal Bovine Serum (FBS), Penicillin–Streptomycin mixtures (Pen–Strep) and trypsin–EDTA solution were purchased from HyClone Laboratories, Inc. 2′,7′-Dichlorodihydrofluorescein Diacetate (DCFH-DA), Calcein Acetoxymethyl Ester (Calcein-AM) and propidium iodide (PI) were purchased from Shanghai Mackinlin Biochemical Co., Ltd. All organic solvents were purchased from Sinopharm Chemical Reagent Co., Ltd. Deionized water prepared with an Aquapro System (18 MΩ) was employed in all experiments.

### Characterizations

^1^H NMR spectra were recorded on a Bruker AVANCE III HD 400 M spectrometer. Spectra were referenced internally using the residual solvent resonance relative to SiMe_4_. MALDI-TOF mass spectra were taken on a Bruker BIFLEX III with alpha-cyano-4-hydroxycinnamic acid as matrix. Elemental microanalyses (EA) were performed with an Elementar Vario EL Cube Elemental Analyzer. The UV-vis absorption spectra were recorded with a UV-2600 Jingdao spectrophotometer. Fluorescence spectra were obtained with FLS-920 Edinburgh Fluorescence Spectrometer with a Xenon lamp. Transmission electron microscopy (TEM) was recorded on a Hitachi HT7700. Electron microscope at an operating voltage of 120 kV. Dynamic Light Scattering (DLS) and zeta potentials were obtained on a Malvern Zetasizer Nano ZS90 with water as solvent at 25 °C. Confocal fluorescence imaging studies were performed with a TCS SP5 confocal laser scanning microscopy (Leica Co., Ltd. Germany) with an objective lens (×20).

### Singlet oxygen generation

Singlet oxygen generation measurement was carried out by modified method using 1,3-diphenylisobenzofuran (DPBF) as capture agent.^19,20^ 62 μL of DPBF (DMF, 1.33 mM) and 2 mL of HSA-α-Pc or HSA-β-Pc aqueous solution (20 μM Pc equiv.) were mixed in a quartz cuvette and irradiated under a 808 nm laser (1.5 W cm^−2^). The absorbance intensity of DPBF at 420 nm was recorded at 1 min intervals. The rate of singlet oxygen generation was determined from the reduced absorbance intensity over time. For the control experiments, DPBF absorption was also recorded for negative comparison at the same conditions in the absence of photosensitizer.

### Photothermal activity

Aqueous solution (1.0 mL) of the Pc NP sample was put in a quartz cuvette and irradiated with an 808 nm laser for 15 min. Pure water was used as a control group. A thermocouple probe with a digital thermometer was used to measure the temperature every 10 s. The photothermal conversion efficiency (*η*) was calculated according to the reported method (eqn (1)–(3)):^21,22^1
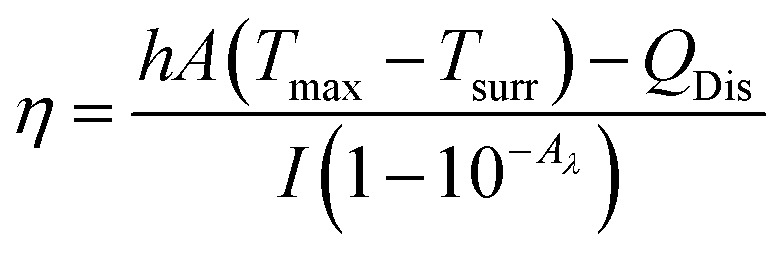
2
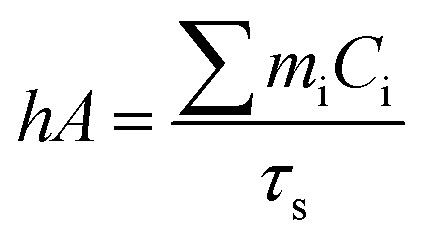
3
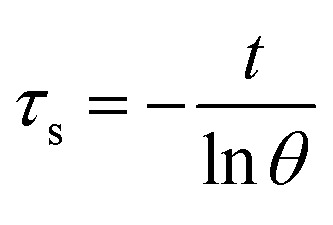
where *hA* are the heat transfer coefficient and surface area of the cuvette cell, respectively. *T*_surr_ and *T*_max_ are initial and final temperature of the solution. *Q*_Dis_ represents the heat dissipation of solvent (water), which was measured independently and found to be 0.014 W. *I* is incident laser power (1.18 W), and *A*_*λ*_ is the absorbance at 808 nm. *m* and *C* are the mass (1 g) and heat capacity (4.2 J g^−1^) of water, respectively. *τ*_s_ is the time constant of sample system. *θ* is the dimensionless driving force and *t* is time.

### Cell culture

Human breast adenocarcinoma cell line (MCF-7 cells) were provided by Institute of Basic Medicine, Shandong Academy of Medical Sciences (Jinan, China). MCF-7 cell lines were cultured in RPMI 1640 supplemented with 10% (v/v) FBS and 1% antibiotics (penicillin/streptomycin, 100 U mL^−1^) at 37 °C under a 5% CO_2_ atmosphere.

### Cytotoxicity assay

The dark cytotoxicity of HSA-α-Pc and HSA-β-Pc NPs as well as their photocytotoxicity were evaluated by 3-(4,5-dimethylthiazol-2-yl)-2,5-diphenyltetrazolium bromide (MTT) assay. Cells were plated at a density of 1 × 10^4^/well in a 96-well cell culture plate at 37 °C under 5% CO_2_ atmosphere overnight. The cells were treated with increasing concentrations (0, 5, 10, 20 μM Pc equiv.) of HSA-α-Pc or HSA-β-Pc NPs (100 μL per well) as treatment group, treated with free RPMI 1640 (100 μL per well) as control group for 24 h at 37 °C under a 5% CO_2_ atmosphere and incubated for 2 h. After the removal of samples, cells were transferred into fresh media and then irradiated by the 808 nm laser at a power density of 1.5 W cm^−2^ for 10 min. The dark cytotoxicity (without irradiation) was monitored without irradiation at the same time as control. The cells were then incubated at 37 °C for additional 24 h before the MTT assay to determine the cell viabilities.

### Confocal laser scanning microscopy (CLSM)

The MCF-7 cells were seeded in glass-bottomed dishes in 2 mL of RPMI 1640 medium. The cells were treated with samples HSA-α-Pc or HSA-β-Pc NPs (both at 20 μM, Pc equiv.) at 37 °C. The cell culture medium was removed, and cells were washed with ice-cold PBS buffer (pH = 7.4) for two times before fixed with fresh 4.0% paraformaldehyde (1 mL) for 10 min at room temperature. The fixed cells were again washed with PBS 7.4 for three times before observation by confocal laser scanning microscope excited at 405 nm and monitored at 450–550 nm.

### *In vitro* singlet oxygen generation tests

MCF-7 cells were incubated with samples (20 μM Pc equiv.) for 2 h, stained with 2′,7′-dichlorodihydrofluorescein diacetate (DCFH-DA) (20 μM) for 10 min and then washed with PBS 7.4. Before and after irradiation with an 808 nm laser (1.5 W cm^−2^) for 5 min, cell images were acquired using a confocal laser scanning microscope. The ROS probe was excited at 488 nm and monitored at 490–590 nm, laser intensity 20%, Smart Grain at 800 V.

### *In vitro* phototherapy

MCF-7 cells were incubated with samples (20 μM, Pc equiv.) for 2 h and then irradiated with an 808 nm laser at a power density of 1.5 W cm^−2^ for 10 min. After 15 min incubation, both Calcein-AM (calcein acetoxymethyl ester) and PI (propidium iodide) were used to co-stain the cells to determine the phototherapy effect of the samples by a laser scanning confocal microscope by using a green channel for live cells (*λ*_ex_ = 488 nm, 500–550 nm), and a red channel for dead cells (*λ*_ex_ = 543 nm, 600–650 nm).

### *In vivo* antitumor therapy

Nude mice (BALB/c-JGpt-Foxn1^nu^/Gpt, male, aged 5 weeks, ∼20 g) were purchased from the Beijing Vital River Laboratory Animal Technology Co., Ltd. Animal experiments were reviewed and approved by the Ethics Committee of Shandong Normal University (Jinan, P. R. China). All the animal operations complied with Chinese government relevant guidelines and regulations for the care and use of experimental animals.

MCF-7 cancer cells (10^6^ cells) suspended in DPBS (100 μL) were subcutaneously injected into the flanks of each mice to establish MCF-7 xenograft model. Length (*L*) and width (*W*) of the tumor were determined by digital calipers. The tumor volume (*V*) was calculated by the formula *V* = 1/2 × *L* × *W*^2^. When the tumor size reached ∼150 mm^3^, the nude mice bearing MCF-7 tumors (*n* = 24) were randomly distributed into 6 groups. After intratumoral injection (200 μM, Pc equiv., 50 μL), the nude mice were feeding for 1 h, and for the treatment group, light treatment (808 nm laser, 1.5 W cm^−2^, 8 min) was performed on the tumor site. The mice continued to be fed for 10 days. The tumor volume and nude mouse body weight were recorded daily during the experimental period.

### Synthesis of 3/4-butylaminophthalonitrile

3/4-Nitrophthalonitrile (10.0 g, 57.8 mmol), *n*-butylamine (4.6 g, 63 mmol), and triethylamine (11.7 g, 116 mmol) were dissolved in 50 mL dry DMF and stirred at room temperature for 12 h. Then, the mixture was poured into 500 mL water, and the precipitate was collected by filtration. The resulting precipitate was recrystallized from dichloromethane and ethanol and gave pure compound as an orange-yellow powder.

#### 3-Butylaminophthalonitrile

Yield, 89% (10.2 g). IR (KBr pellet cm^−1^): 3374(vs), 3094(w), 2955(s), 2869(m), 2224(s), 1956(w), 1860(w), 1763(w), 1613(vs), 1516(s), 1344(s), 1205(w), 1065(w), 775(s), 732(w), 571(w), 463(w). ^1^H NMR (400 MHz, CDCl_3_, 25 °C, TMS, ppm): 7.47–7.43 (t, 1H,–C_6_H_3_–), 7.01–6.99 (d, 1H,–C_6_H_3_–), 6.91–6.89 (d, 1H, –C_6_H_3_–), 4.81 (s, 1H, –NH–), 3.25–3.21 (t, 2H, –CH_2_–), 1.70–1.63 (m, 2H, –CH_2_–), 1.48–1.43 (m, 2H, –CH_2_–), 1.00–0.96 (t, 3H, –CH_3_).

#### 4-Butylaminophthalonitrile

Yield, 86% (9.8 g). IR (KBr pellet cm^−1^): 3363(vs), 3084(w), 2955(s), 2859(m), 2224(s), 1945(w), 1623(vs), 1526(s), 1473(m), 1344(m), 1258(m), 1086(w), 850(s), 732(w), 636(w), 539(m). ^1^H NMR (400 MHz, CDCl_3_, 25 °C, TMS, ppm): 7.51–7.49 (d, 1H,–C_6_H_3_–), 6.83 (s, 1H,–C_6_H_3_–), 6.75–6.73 (d, 1H, –C_6_H_3_–), 5.34 (s, 1H, –NH–), 3.19–3.16 (t, 2H, –CH_2_–), 1.68–1.60 (m, 2H, –CH_2_–), 1.45–1.40 (m, 2H, –CH_2_–), 1.00–0.96 (t, 3H, –CH_3_).

### Synthesis of metal-free tetra(butylamino)phthalocyanines

Li (28 mg, 4.0 mmol) in extra dry *n*-butanol (2.0 mL) was heated to 90 °C for 1 h under N_2_ atmosphere and then 3/4-butylaminophthalonitrile (80 mg, 0.4 mmol) was added. The resulting mixture was heated to reflux for 4 h. After being cooled to room temperature, the volatiles were evaporated *in vacuo* and the residue was chromatographed on a neutral alumina column with dichloromethane : tetrahydrofuran (1 : 1) as eluent. Repeated chromatography followed by recrystallization from dichloromethane and methanol and gave pure tetra(butylamino)phthalocyanine compounds as dark powder.

#### 1(4),8(11),15(18),22(25)-Tetra(butylamino)phthalocyanine (α-Pc)

Yield, 25% (20 mg). UV-vis [*λ*_max_ (nm) (log(*ε*), M^−1^ cm^−1^)]: CHCl_3_, 326 (4.83), 516 (4.10), 708 (4.68), 786 (5.08). IR (KBr pellet cm^−1^): 3309(w), 3078(w), 2963(vs), 2919(vs), 2574(w), 1909(w), 1608(vs), 1510(s), 1466(m), 1341(vs), 1218(m), 1164(m), 1093(m), 1032(s). ^1^H NMR (400 MHz, CDCl_3_/[D_6_]DMSO (6 : 1), 25 °C, TMS, ppm): 7.80–6.58 (–Pc_C_6_H_3__–), 3.64–3.38 (4H, –NH–), 2.20–1.78 (–CH_2_–), 1.18–0.79 (–CH_2_CH_2_CH_3_), −1.95 (–Pc_NH_–). MALDI-TOF/MS: *m*/*z* calcd for C_48_H_54_N_12_ [M^+^] 798.46; found 798.42. Anal. calcd For C_48_H_54_N_12_ (%): C, 72.15; H, 6.81; N, 21.04, found C, 72.07; H, 6.32; N, 21.61.

#### 2(3),9(10),16(17),23(24)-Tetra(butylamino)phthalocyanine (β-Pc)

Yield, 25% (20 mg). UV-vis [*λ*_max_ (nm) (log(*ε*), M^−1^ cm^−1^)]: CHCl_3_, 340 (4.99), 447 (4.63), 667 (4.78), 741 (5.07). IR (KBr pellet cm^−1^): 3402(m), 3303(w), 2945(s), 2876(m), 2210(m), 1614(vs), 1505(s), 1346(m), 1276(w), 1117(m), 1008(m), 829(w), 760(m). ^1^H NMR (400 MHz, [D_6_]DMSO, 25 °C, TMS, ppm): 8.95–8.82 (4H, –Pc_C_6_H_3__–), 8.37–8.20 (4H, –Pc_C_6_H_3__–), 7.38–7.34 (4H, –Pc_C_6_H_3__–), 6.96–6.79 (4H, –NH–), 3.44–3.30 
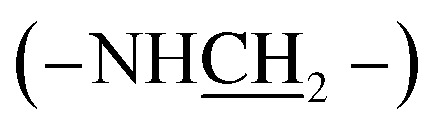
, 1.81–0.93 (28H, –CH_2_CH_2_CH_3_). MALDI-TOF/MS: *m*/*z* calcd for C_48_H_54_N_12_ [M^+^] 798.46; found 798.75. Anal. calcd for C_48_H_54_N_12_ (%): C, 72.15; H, 6.81; N, 21.04, found C, 72.08; H, 6.42; N, 21.50.

### Synthesis of HSA-α-Pc and HSA-β-Pc NPs

2.0 mg of α-Pc was dispersed in 2.0 mL of dimethylacetamide (DMAC). Then, 60 μL of the α-Pc/DMAC solution was mixed with 2.0 mL of the human serum albumin (HSA) aqueous solution (1 mg mL^−1^) and stirred for 1 h. After repeating the above-mentioned procedure for five times, the HSA-α-Pc NP was obtained by centrifugation together and washed with water for three times before storing in water (2.0 mL). 200 μL HSA-α-Pc NP aqueous solution and 200 μL H_2_O were added to the mixture solution of 200 μL DMSO and 2.0 mL chloroform and then 200 μL DMSO was added to the aqueous solution. Five minutes later, all the above mixtures were shaken until the phase of water changed from the dark-green to colorless. The concentrate of α-Pc in the above organic solution was assessed based on the UV standard working curves and then the amounts of α-Pc in NPs aqueous solution could also be calculated. The HSA-β-Pc NP was prepared in the same way as that of HSA-α-Pc NP, in which β-Pc was used instead of α-Pc. In the same way, the amounts of β-Pc in NP aqueous solution used below was assessed based on the UV standard working curves.

## Results and discussion

### Synthesis and characterization of HSA-α-Pc and HSA-β-Pc NPs

**Fig. 1:**
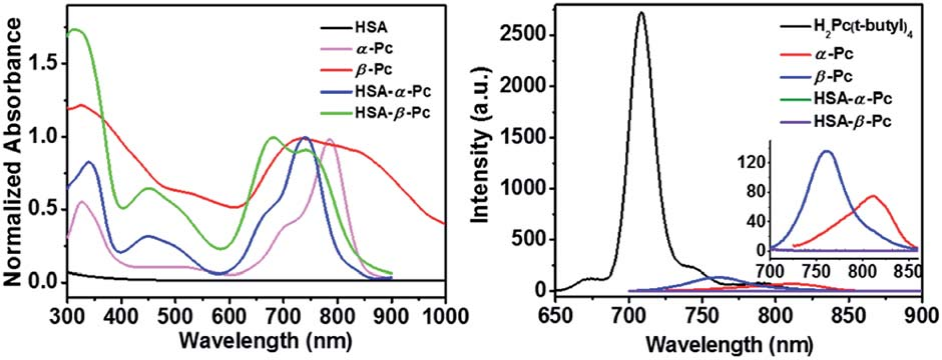
Left: Comparison in the electronic absorption spectra of α-Pc (chloroform), β-Pc (chloroform), HSA-α-Pc NPs (water), and HSA-β-Pc NPs (water), as well as HSA in aqueous solution (1.0 mg mL^−1^). Right: Fluorescence spectra of H_2_Pc(*t*-butyl)_4_ (0.5 μM, excited at 630 nm), α-Pc (0.5 μM, excited at 680 nm), and β-Pc (0.5 μM, excited at 680 nm) in chloroform as well as HSA-α-Pc NPs and HSA-β-Pc NPs (20 μM Pc equiv., excited at 680 nm) in aqueous solutions.

As shown in Scheme 1, α-Pc and β-Pc were synthesized based on 3-butylaminophthalonitrile and 4-butylaminophthalonitrile, respectively (for details see the ESI, Fig. S1–S3 and Table S1†).^23–25^ The obtained α-Pc and β-Pc displayed a broad Q band at 786 and 741 nm, respectively (Fig. 1). Compared to their pristine H_2_Pc (Q band = 672 nm),^26^ the large red-shifts of 114 and 69 nm in their Q-band regions which was caused by the strong electron-donating dibutylamino group and p–π conjugation between the N atom and the central phthalocyanine chromophore. As is known, the prime window for the light delivery through tissue is 700–900 nm, in which light absorbance and scattering from endogenous absorbers such as oxy- and deoxyhemoglobin, water and lipids are minimized.^11^ Therefore, both α-Pc and β-Pc with increased conjugation length could be useful candidates to construct Pc-based nanomaterials for the NIR phototherapy.^13^

On the other hand, Pcs are known to be poor biocompatibility due to their notorious aggregating property. For addressing such an issue, human serum albumin (HSA), which has been demonstrated to be a useful drug carrier to sequester inorganic oxide or organic molecules,^27,28^ was selected to prepare the HSA-involved α-Pc and β-Pc NPs. As is shown in Scheme 1, after separately combining the HSA aqueous solution with the dimethylacetamide (DMAC) solutions of α-Pc and β-Pc, the HSA-Pc NPs of HSA-α-Pc and HSA-β-Pc were readily produced (for details see the ESI†). The generated HSA-α-Pc and HSA-β-Pc NPs were collected by centrifugation and washed with water for three times before storing in water (Fig. S4†). The uploaded phthalocyanine amount in the obtained Pc NPs was easily determined by the UV standard working curves after being extracted with organic solution (for details see the ESI, Fig. S5 and S6†).

**Fig. 2:**
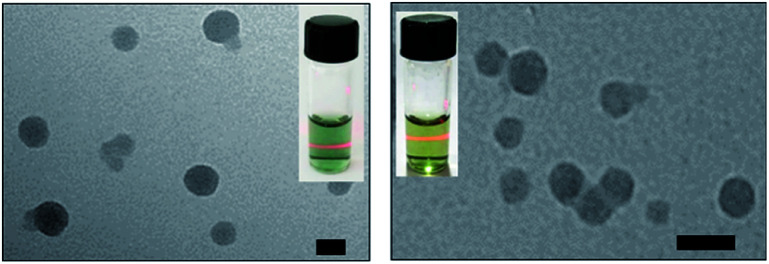
TEM images of HSA-α-Pc NPs (left) and HSA-β-Pc NPs (right), scale bars: 50 nm. The insets present the picture of their Tyndall phenomenon in aqueous solution, which further supports the TEM and DLS analysis.

As shown in Fig. 2, the transmission electron microscopy (TEM) images of the HSA-α-Pc and HSA-β-Pc NPs indicated that they all featured spherical particles with the diameter of *ca.* 60 and 30 nm respectively, which was further supported by the dynamic light scattering (DLS) analysis (centered at 75 and 35 nm respectively, Fig. S4†). The slight difference in size should result from the solvation effect depending on the different measurement.^29^ The suitable size of 35–75 nm with negatively charged surface would enable the HSA-α-Pc and HSA-β-Pc NPs to accumulate in the tumor sites through the enhanced permeability and retention (EPR) effect.^30^ For biological application, the stability of HSA-α-Pc and HSA-β-Pc NPs in ultrapure water, PBS (pH = 6.5 and 7.4), and cell culture media (RPMI 1640) was examined. As shown in Fig. S4,† they all displayed excellent dispersity and stability in all media without significant macroscopic aggregates within five days. Besides, the measured *ζ* potentials of the HSA-α-Pc and HSA-β-Pc NPs are *ca.* −15.4 and −17.8 mV respectively (Fig. S7†), which also implied that these colloidal systems are stable and biocompatible.

It is different from α-Pc and β-Pc monomers, the HSA-α-Pc and HSA-β-Pc NPs in water displayed the broadened absorption bands at 620–960 nm and 590–830 nm, respectively (Fig. 1). Moreover, their maxima adsorptions were blue-shifted with respect to the α-Pc and β-Pc monomers in chloroform (Fig. 1), suggesting that they might feature a face-to-face stacking mode in the HSA-based NPs.

It is known that phthalocyanine fluorescence emission included S_1_ (Q band, lowest state) and S_2_ (Soret, upper excited state) emissions.^31–33^ As shown in Fig. 1, the fluorescent emission spectra of both α-Pc and β-Pc in chloroform indicated that their S_1_ fluorescent emission at 812 and 761 nm were largely quenched. They all possessed very low fluorescence quantum yields (*Φ*_α-Pc_ = 0.0045, *Φ*_β-Pc_ = 0.0049) with respect to the reference of tetrakis(*tert*-butyl)phthalocyanine H_2_Pc(*t*-butyl)_4_ (*Φ* = 0.77, chloroform), which is mainly due to the increased nonradiative decay rate associated with the p–π conjugation between the substituted N atoms and the central phthalocyanine chromophore. The same phenomenon was previously observed in tetrakis(dibutylamino)phthalocyanine H_2_Pc[N(C_4_H_9_)_2_]_4_.^24^ Notably, after formation of the HSA-Pc NPs, the S_1_ fluorescence emission of HSA-α-Pc (*Φ* = 0) and HSA-β-Pc (*Φ* = 0) NPs were completely quenched due to the ACQ effect (Fig. 1). Some previous studies have shown that the S_1_ fluorescence emission quenching of Pcs would significantly improve their light-to-heat conversion.^8,11,12^ Therefore, HSA-α-Pc and HSA-β-Pc herein are expected to be the potential photothermal materials for phototherapy of cancer under NIR laser irradiation.

### Photoproperties of HSA-α-Pc and HSA-β-Pc NPs

As shown above, the S_1_ fluorescence emission of the HSA-α-Pc and HSA-β-Pc NPs in water were significantly suppressed, so their excellent photothermal performance was expected.^34^

**Fig. 3:**
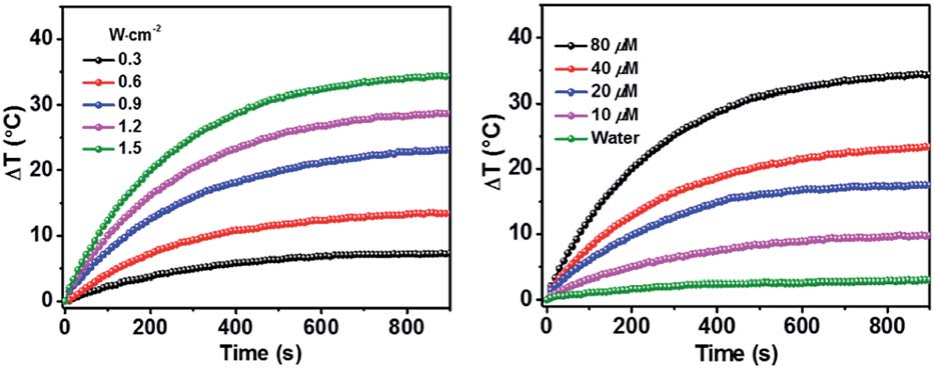
Photothermal property of HSA-α-Pc NPs in aqueous solution under 808 nm laser irradiation. Left: Light intensity-dependent temperature increase at the concentration of 80 μM (Pc equiv.). Right: Concentration-dependent temperature increase with a 0.9 W cm^−2^ laser intensity.

Fig. 3 showed that the photothermal behaviour of HSA-α-Pc NP was concentration and light intensity dependent, and the highest temperature increment (Δ*T*) of 34 °C was observed at a concentration of 80 μM (Pc equiv.) under 808 nm laser irradiation at 1.5 W cm^−2^. In contrast, only *ca.* 3 °C temperature increase of pure water was detected in the absence of HSA-α-Pc NP. This suggested that HSA-α-Pc was a highly efficient photothermal conversion species which was able to convert light energy into heat in an effective and quick way even irradiated by laser at 808 nm. To determine the photothermal efficiency of HSA-α-Pc, its heating and cooling curve in water was measured (Fig. S8†). According to the obtained data, the light-to-heat conversion efficiency *η* was calculated to be 56%, which is higher than those of HSA-FePc (44%, 671 nm),^35^ DBCO-ZnPc-LP (44%, 808 nm),^36^ O_2_@PFH@HMoS_*x*_-HSA/AlPc (40%, 670 nm),^37^ 4OCSPC/F127 micelles (47%, 808 nm).^38^ After six cycles, the photothermal heating capability for the HSA-α-Pc NP at 20 μM (Pc equiv.) under 808 nm laser irradiation (1.5 W cm^−2^) remained robust with Δ*T* up to 17 °C, demonstrating its excellent photostability. Similarly, the HSA-β-Pc NP also possessed an excellent photothermal conversion efficiency with *η* value of 54% and temperature increase of 17 °C under the same conditions (808 nm, 1.5 W cm^−2^, 20 μM Pc equiv.) (Fig. S9†). The high photothermal performance and high NIR photostability exhibited by HSA-α-Pc and HSA-β-Pc make them promising photothermal agents for phototherapy of cancer.

**Fig. 4:**
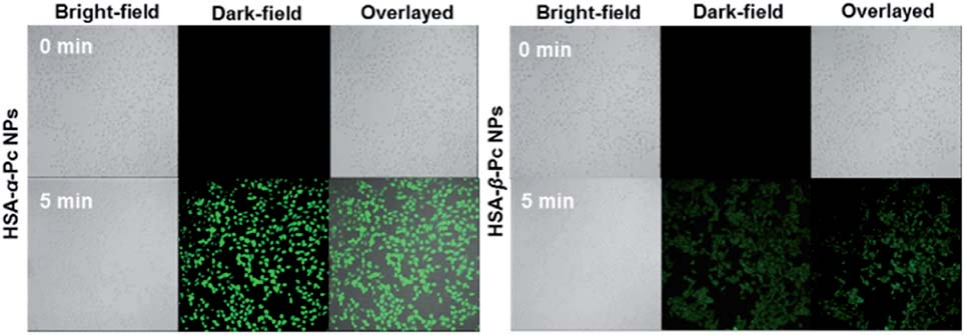
ROS generation induced by HSA-α-Pc and HSA-β-Pc (both at 20 μM Pc equiv.) in MCF-7 cells in the presence of laser irradiation for 5 min (808 nm, 1.5 W cm^−2^).

In addition, the single oxygen generating ability of HSA-α-Pc and HSA-β-Pc under NIR light (808 nm) illumination was investigated using DPBF as a detector by monitoring oxidation of DPBF at 420 nm. Compared to HSA-β-Pc, HSA-α-Pc exhibited more efficient singlet oxygen production (Fig. S10†), which might be caused by the different location of peripheral group in Pc.^39^ The singlet oxygen generation of the HSA-α-Pc and HSA-β-Pc NPs in cells upon 808 nm laser irradiation were also examined, and the result indicated that HSA-α-Pc also induced higher singlet oxygen generation in MCF-7 cells than that of HSA-β-Pc (Fig. 4).

### *In vitro* and *in vivo* photothermal therapy

For examination of the cellular biocompatibility and membrane permeability of HSA-α-Pc and HSA-β-Pc NPs, cell imaging experiments were carried out against MCF-7 cells by confocal laser scanning microscopy (CLSM). As is shown, both HSA-α-Pc and HSA-β-Pc NPs displayed a weak S_2_ emission of Pc^31–33^ with a maximum band at around 469 nm upon 405 nm irradiation (Fig. S11†). After they (both at 20 μM, Pc equiv.) separately incubated with MCF-7 cells for 2 h, MCF-7 cells were visualized by the green emission from Pc (*λ*_ex_ = 405 nm). In addition, we noticed that the green (440–480 nm) luminescence mainly located in the cytoplasm, demonstrating that the HSA-α-Pc and HSA-β-Pc NPs could readily pass across the tumour cell membrane into the cytoplasm (Fig. S12†).

**Fig. 5:**
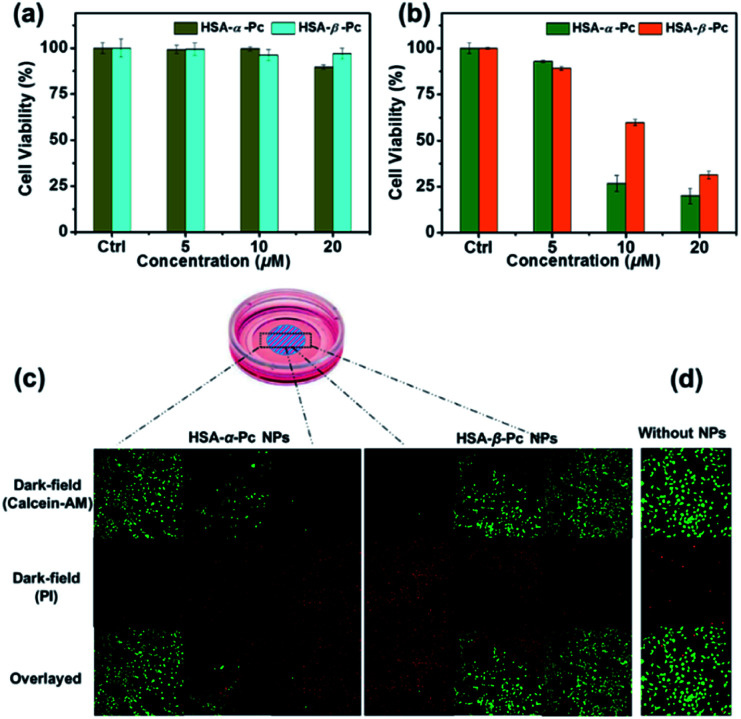
*In vitro* cytotoxicities of HSA-α-Pc and HSA-β-Pc NPs against MCF-7 cells before (a) and after (b) being irradiated with 808 nm laser at a power density of 1.5 W cm^−2^ for 10 min. (c) A sketch map of the cell culture dish after incubation with Pc NPs. The cyan circle with shadow shows the laser spot. (d) Confocal images of Calcein-AM (green, live cells) and PI (red, dead cells) co-stained MCF-7 cells incubated without and with HSA-α-Pc and HSA-β-Pc NPs (both at 20 μM, Pc equiv.) before and after exposed to irradiation for 10 min (808 nm, 1.5 W cm^−2^).

In addition, the *in vitro* phototoxicity of HSA-α-Pc and HSA-β-Pc NPs were tested by using the standard 3-(4,5-dimethyl-2-thiazolyl)-2,5-diphenyltetrazolium bromide (MTT) assays against MCF-7 cells at various concentrations (0–20 μM, Pc equiv.) under irradiation at a power density of 1.5 W cm^−2^ for 10 min. As shown in Fig. 5a, before laser irradiation, both HSA-α-Pc and HSA-β-Pc NPs could maintain more than 90% cell viability, indicating that they were biocompatible with negative dark cytotoxicity. As shown in Fig. 5b, upon 808 nm laser irradiation (1.5 W cm^−2^, 10 min), HSA-α-Pc showed a high phototoxicity of *ca.* 80% at the concentration of 20 μM (Pc equiv.) for the tested cell line, which was higher than that of HSA-β-Pc (*ca.* 69%) at the same concentration (20 μM, Pc equiv.). Their superior therapeutic effect was also directly demonstrated by a live/dead cell co-staining study. As shown in Fig. 5c, only the cells exposed to laser irradiation (within the cyan shadow) were killed, and a clear demarcation line between live cells (green) and dead cells (red) was observed in both cell lines (Fig. 5d). Owing to the similar photothermal heating capability of HSA-α-Pc and HSA-β-Pc (20 μM, Pc equiv.) under 808 nm laser irradiation mentioned above, the higher phototoxicity exhibited by HSA-α-Pc was clearly attributed to the fact that HSA-α-Pc generated more single oxygen and could kill cancer cells more efficiently *via* synergistic PDT/PTT under a single NIR laser irradiation.

Recently, some very impressive works of Pc-nanomaterials for PTT/PDT phototherapy have been reported, which are summarized in Table 1.^9,10,13,37,40–42^ Compared to the reported Pc-based NPs, the phototherapy treatment of HSA-α-Pc in living cells met a single NIR light source (808 nm), high light-to-heat conversion efficiency *η* (56%) and tiny Pc amount (20 μM) for less than 20% cell viability. So, HSA-α-Pc herein is in a strong position among the Pc-based phototherapy nanomaterials.

**Table 1:** Summary of recent typical Pc-nanomaterials for PTT/PDT synergistic phototherapy in living cells by a single light source

Sample	Light source (W cm^−2^)	*η* (%)	PS (μM)	MTT (%)	Ref.
Pc@HSNs	730 nm (1.5)	37	∼575	30	9
NanoPcTB	655 nm (2.5)	—	6	20	10
ZnPc NW	808 nm (3.0)	—	∼205	50	13
O2@PFH@HMoS_*x*_-HSA/AlPc	670 nm (1.0)	40	∼255	25	37
GR-TSCuPc	650 nm (3.0)	—	∼15	40	40
ZnPc NPs	650 nm (0.7)	31	20	∼15	41
SWNHs−TSCuPc	650 nm (3.0)	—	∼15	15	42
HSA-β-Pc	**808 nm (1.5)**	**54**	**20**	**31**	**This work**
HSA-α-Pc	**808 nm (1.5)**	**56**	**20**	**20**	**This work**

**Fig. 6:**
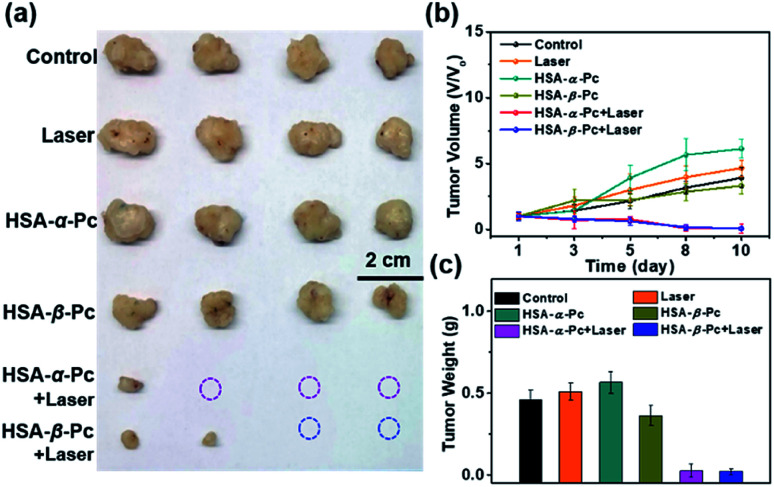
Nude mice bearing MCF-7 tumors (*n* = 24) were randomly distributed into six groups when the tumor size reached ∼140 mm^3^. Light treatment was performed on the tumor site using an 808 nm laser (1.5 W cm^−2^, 8 min) for therapy. (a) Photographs of tumor tissue obtained after treatment. (b) Tumor volume of the nude mice in various groups during the treatment. (c) Tumor weight obtained after treatment. All data are presented as the mean ± SD (*n* = 4).

To further investigate the phototherapy of HSA-α-Pc and HSA-β-Pc NPs, the *in vivo* antitumor effects of Pc NPs were evaluated by an MCF-7 xenograft model. Twenty-four nude mice bearing tumors were randomly divided into six groups (Fig. 6 and S13†). For the group of laser only, the tumor volume increased rapidly, and there was no difference from the control group. After the intratumor injection of HSA-α-Pc and HSA-β-Pc NPs (200 μM, Pc equiv., 50 μL) but both without light treatment, the tumor volume increased rapidly also similar to the control group, respectively. When light treatment using an 808 nm laser (1.5 W cm^−2^) for 8 min, their therapy eradicated almost all of the tumor tissues, and scabs were exfoliated on days 8–10 without obvious signs of recurrence, indicating the metal-free tetra(butylamino)phthalocyanine Pc-based nanomaterials could be used as phototherapy agents for deep tumor treatment.

## Conclusions

In conclusion, the first of its kind, we developed a very facile approach to fabricate metal-free Pc NPs (HSA-α-Pc and HSA-β-Pc) by assembling of the butylamino-decorated metal-free Pcs with HSA under ambient conditions. The generated HSA-Pc NPs possess NIR absorption (750–900 nm), low dark cytotoxicity, enhanced photostability and good membrane permeability as well as highly efficient light-to-heat energy conversion, which caused the obtained Pc nanomaterials to be the potential synergistic PTT/PDT agents for deep tumor treatment. We expect this approach would be viable for the fabrication of many more new Pc-based metal-free nano agents for PDT/PTT synergistic phototherapy upon a single NIR light source. Moreover, the blood circulation and biodistribution of these HSA-Pc NPs need to be examined in the future for clinical applications.

## Conflicts of interest

There are no conflicts to declare.

## Supplementary Material

RA-010-D0RA03898A-s001
