# Supplementary material for: Near-infrared and metal-free tetra(butylamino)phthalocyanine nanoparticles for dual modal cancer phototherapy
Source: RSC Adv. 2020 Jul 9;10(43):25958–65. doi: 10.1039/d0ra03898a (PMC9055336; doi:10.1039/d0ra03898a)
Supplement: RA-010-D0RA03898A-s001 [file RA-010-D0RA03898A-s001.pdf]

Electronic Supplementary Material (ESI) for RSC Advances.
This journal is © The Royal Society of Chemistry 2020

**Supporting Information for**

# Near-infrared and metal-free tetra(butylamino)phthalocyanine nanoparticles for dual modal cancer phototherapy

Ying-Jie Wu,‡[a] Fan-Hong Lv, ‡[a] Jing-Lan Kan,*[a] Qun Guan,[a] Anqi Xue,[b] Quanbo Wang,[b] Yan-An Li,[a] and Yu-Bin Dong*[a]

[a] College of Chemistry, Chemical Engineering and Materials Science, Collaborative Innovation Center of Functionalized Probes for Chemical Imaging in Universities of Shandong, Key Laboratory of Molecular and Nano Probes, Ministry of Education, Shandong Normal University, Jinan 250014, P. R. China.

[b] Shandong Analysis and Test Center, Qilu University of Technology (Shandong Academy of Sciences), Jinan 250014, P. R. China.

* Corresponding authors.

E-mail addresses: kanjinglan@163.com (J.-L. Kan), yubindong@sdnu.edu.cn (Y.-B. Dong).

‡ Contributed equally

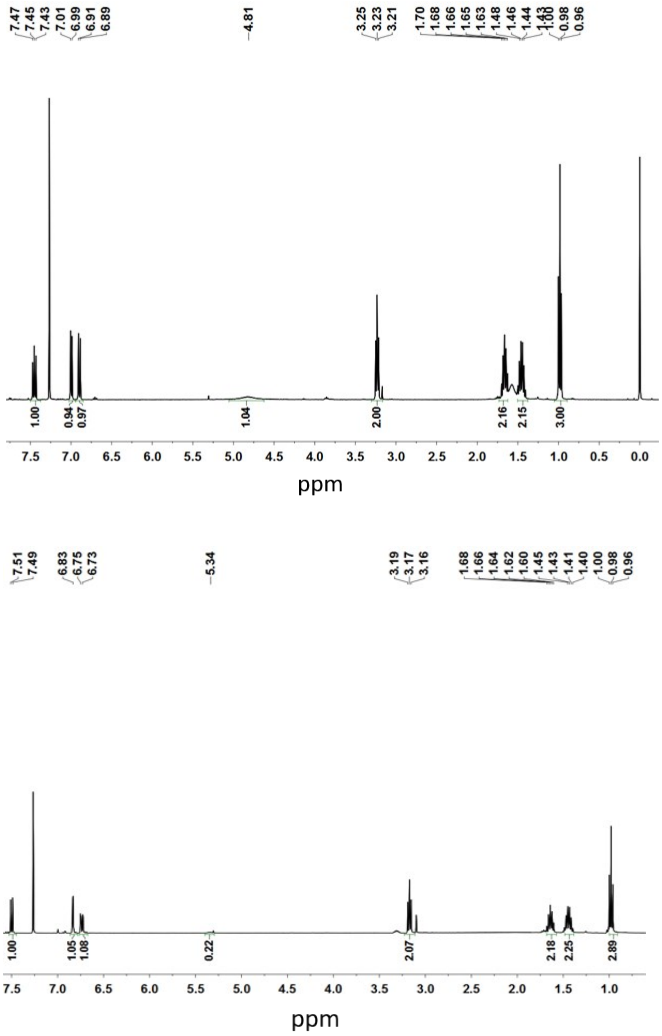


**Fig. S1** $^{1}H$ NMR spectra of 3-butylaminophthalonitrile (top) and 4-butylaminophthalonitrile (bottom) in $CDCl_3$. The signal at 7.26 ppm was attributed to the residual $CHCl_3$.

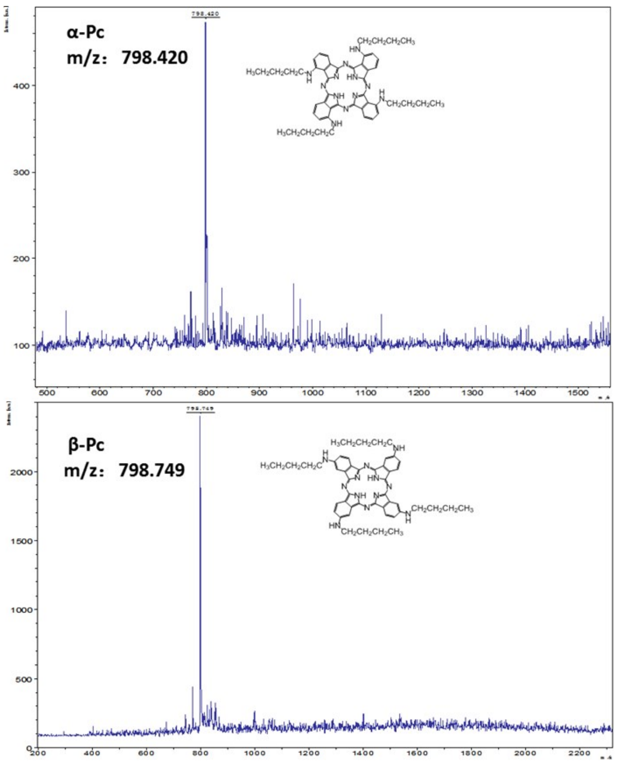


**Fig. S2** MALDI-TOF mass spectra for ***α*-Pc** and ***β*-Pc**.

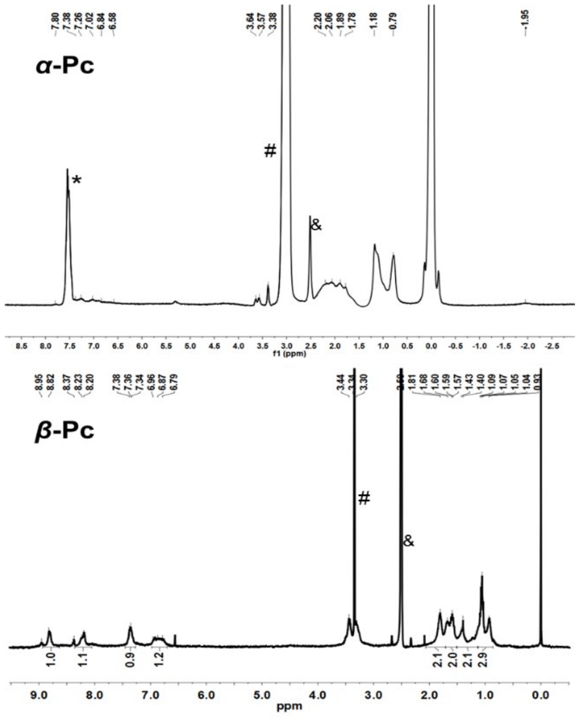


**Fig. S3** $^1$H NMR spectra of ***α*-Pc** (top) in $CDCl_3$/[$D_6$]DMSO (6:1) and ***β*-Pc** (bottom) in [$D_6$]DMSO (with the signals due to residues of $CHCl_3$, $H_2O$ and DMSO denoted as *,$^{\#}$ and $^{\&}$, respectively).

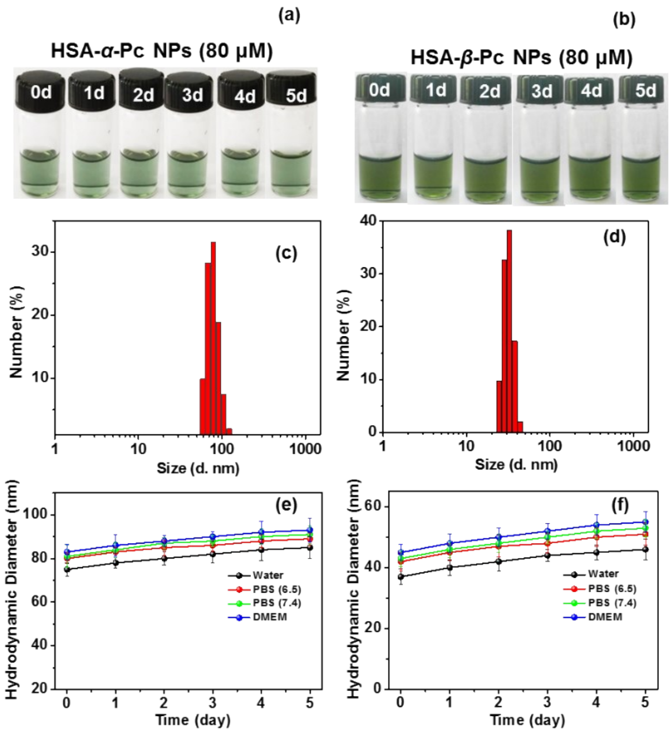


**Fig. S4** Pictures of **HSA-*α*-Pc** NP (a) and **HSA-*β*-Pc** NP (b) in water (both at 80 *μ*M, Pc equiv) after standing for different time. The DLS analysis of **HSA-*α*-Pc** NP (c) and **HSA-*β*-Pc** NP (d) dispersed in aqueous solution. The DLS stability of **HSA-*α*-Pc** NP (e) and **HSA-*β*-Pc** NP (f) in various media for five days, and the data were based on the results of 3 independent experiments.

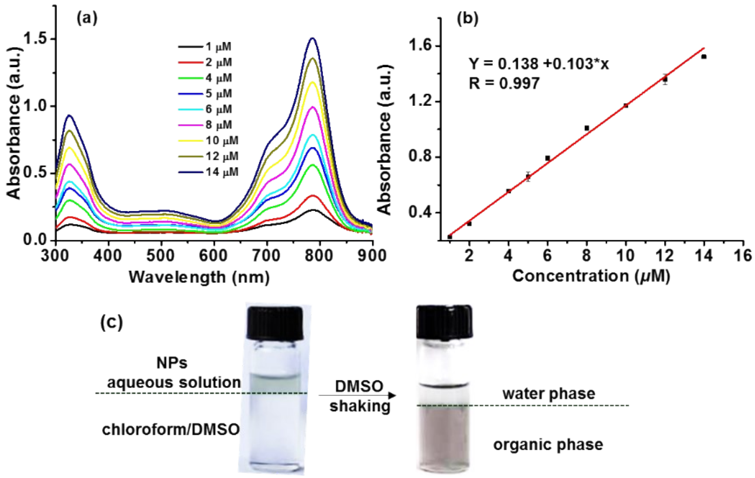


**Fig. S5** (a) The UV-vis spectra of ***α*-Pc** in V($CHCl_3$) :V(DMSO) = 95:5 solution at different concentrations. (b) The calibration curve of ***α*-Pc** at 786 nm. The absorbance data were presented as mean ± SD (*n* = 3). (c) The procedure of calculating the amounts of ***α*-Pc** in NP aqueous solution by using organic solvent extraction.

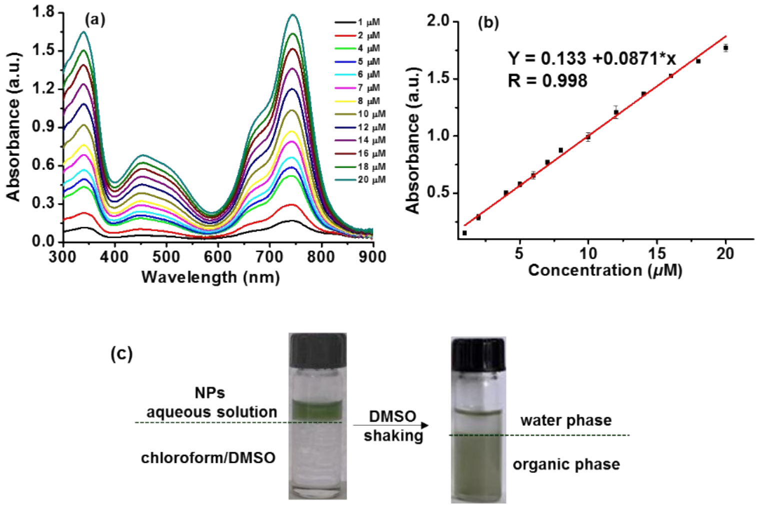


**Fig. S6** (a) The UV-vis spectra of ***β-Pc*** in V($CHCl_3$) : V(DMSO) = 95:5 solution at different concentrations. (b) The calibration curve of ***β*-Pc** at 742 nm. The absorbance data were presented as mean ± SD (*n* = 3). (c) The procedure of calculating the amounts of ***β*-Pc** in NP aqueous solution by using organic solvent extraction.

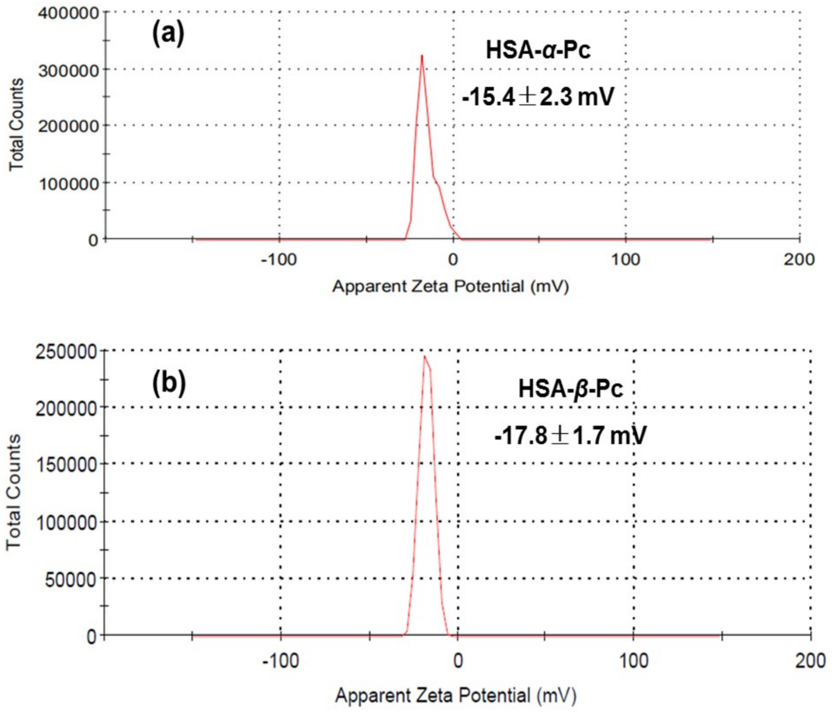


**Fig. S7** The zeta potentials of **HSA-*α*-Pc** NP (a) and **HSA-*β*-Pc** NP (b) in water solution at 25°C (both at 80 $\mu$M, Pc equiv).

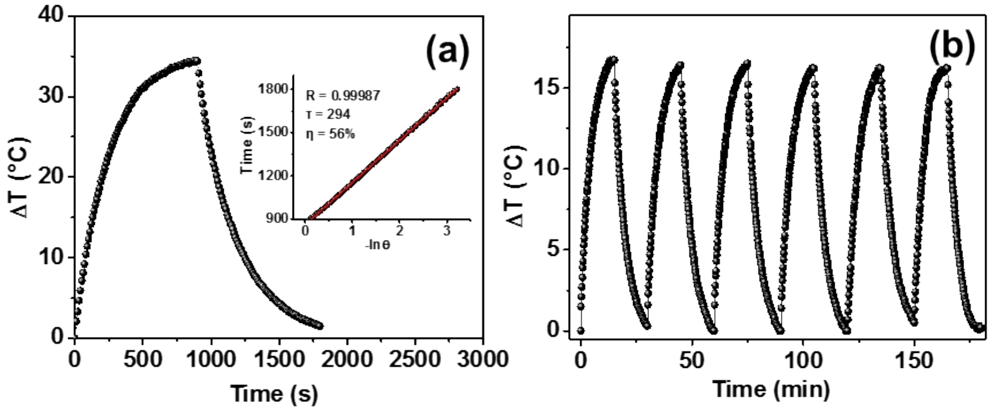


**Fig. S8** Photothermal property of **HSA-*α*-Pc** NP in aqueous solution under 808 nm laser irradiation. (a) Photothermal effect and the laser was switched off after irradiation for 15 min (80 $\mu$M Pc equiv, 1.5 W·cm$^{-2}$). The inset presents the plot of cooling time (15 min) *vs* the negative natural logarithm of the temperature driving force obtained from the cooling stage. (b) Temperature variations for 6 cycles of the (1.5 W·cm$^{-2}$, 15 min per cycle) at the concentration 20 $\mu$M (Pc equiv).

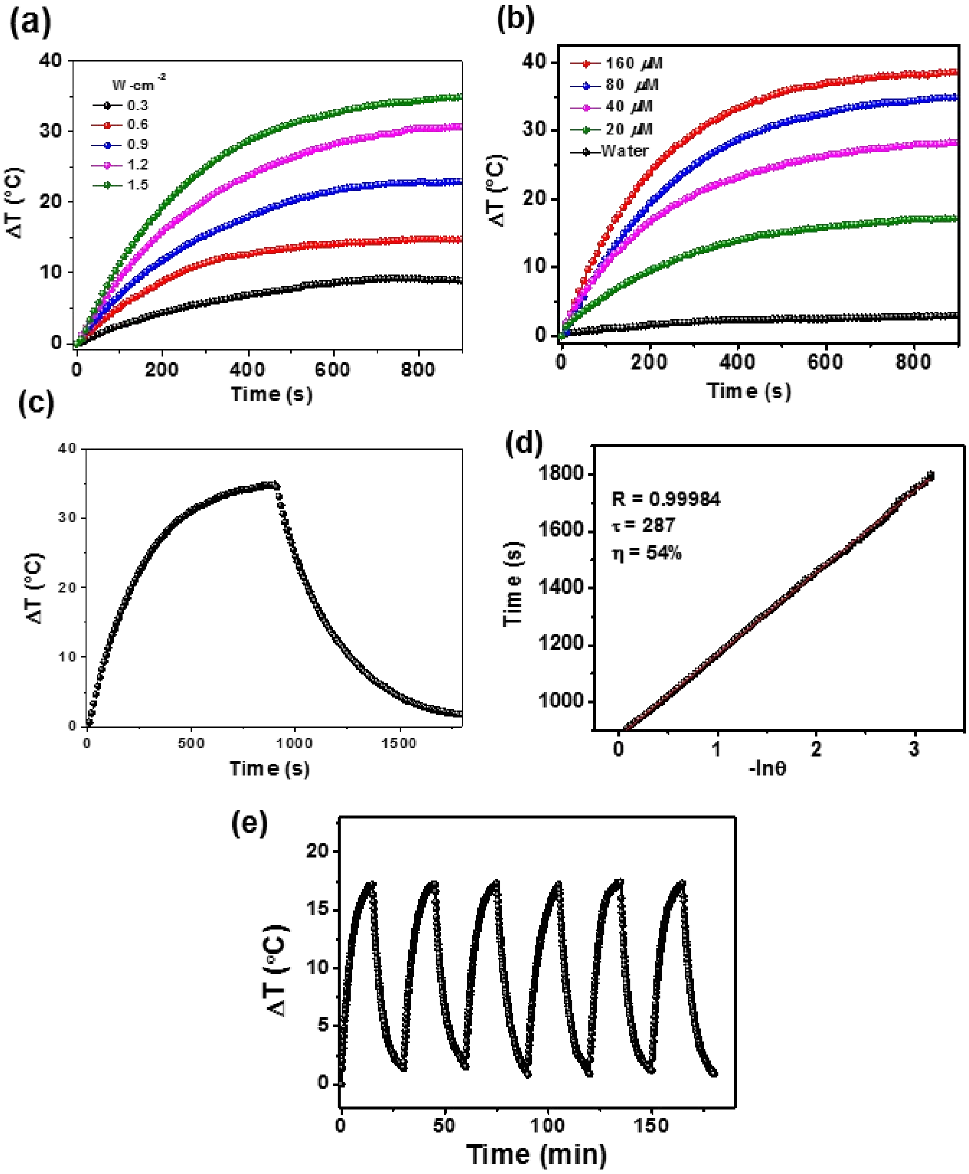


**Fig. S9** Photothermal property of **HSA-*β*-Pc** NP dispersed in water under 808 nm laser irradiation. (a) Temperature elevation in dependence of light intensity at the concentration of 80 μM (Pc equiv). (b) Concentration-dependent temperature elevation with an intensity of 0.9 W·cm$^{-2}$. (c) Photothermal effect and the laser was switched off after irradiation for 15 min (80 μM, Pc equiv., 1.5 W·cm$^{-2}$). (d) Plot of cooling time (15 min) *vs* the negative natural logarithm of the temperature driving force obtained from the cooling stage. (e) Temperature variations for 6 cycles of the (1.5 W·cm$^{-2}$, 15 min per cycle) at the concentration 20 μM (Pc equiv).

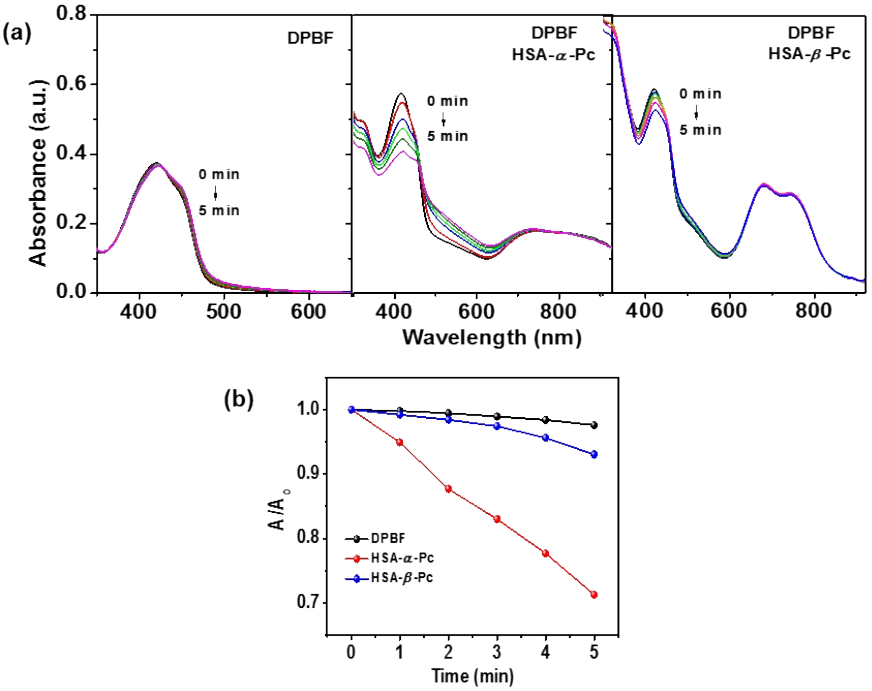


**Fig. S10** Singlet oxygen generation ability of the **HSA-*α*-Pc** and **HSA-*β*-Pc** NPs. (a) Time-dependent UV-vis absorption of DPBF/DMF (1.33 mM, 62 $\mu$L) at 420 nm with **HSA-*α*-Pc** and **HSA-*β*-Pc** (both at 20 $\mu$M Pc equiv, 2.0 mL) in aqueous solution under 808 nm laser irradiation (1.5 W·cm$^{-2}$) for 5 min. (b) Comparison of the decay rate of DPBF, **HSA-*α*-Pc** and **HSA-*β*-Pc**.

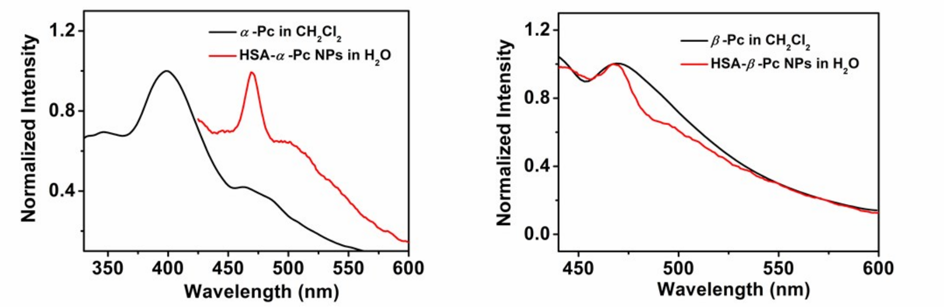


**Fig. S11** Fluorescence spectra of ***α*-Pc** (5 $\mu$M, $CH_2Cl_2$, excited at 300 nm) and ***β*-Pc** (5 $\mu$M, $CH_2Cl_2$, excited at 309 nm) as well as **HSA-*α*-Pc** and **HSA-*β*-Pc** NPs (both at 20 $\mu$M, Pc equiv) in water upon irradiated at 405 nm.

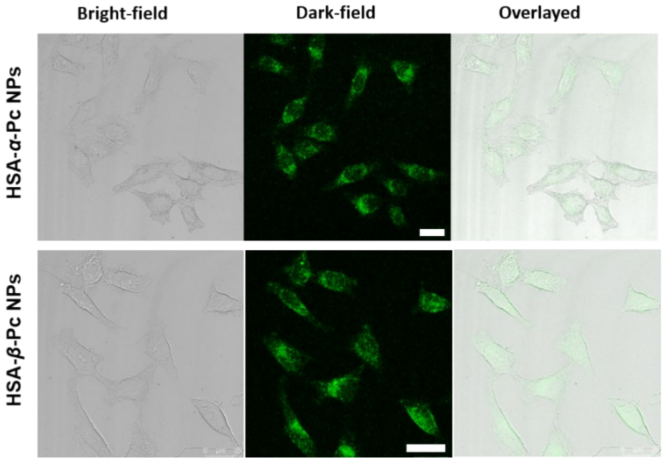


**Fig. S12** *In vitro* CLSM images of MCF-7 cells treated with **HSA-α-Pc** and **HSA-β-Pc** NPs (both at 20 $\mu$M, Pc equiv) for 2 h, using a green channel for phthalocyanine ($\lambda_{ex}$ = 405 nm, 440-480 nm), scale bar 25 $\mu$m.

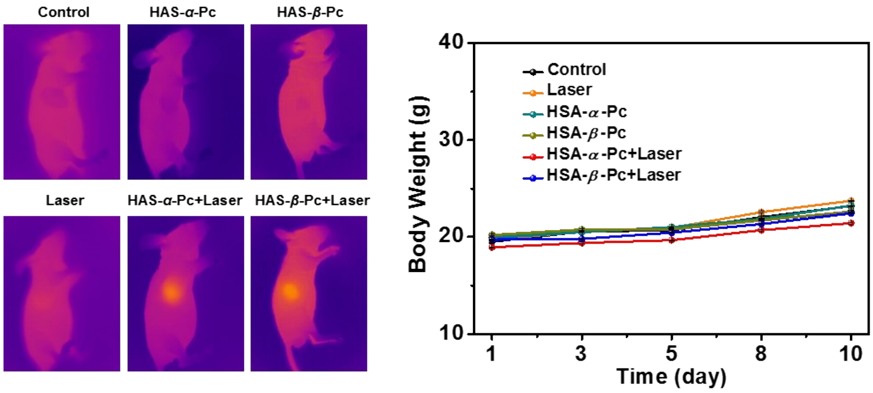


**Fig. S13** (Left) Thermal imageries of the nude mice. (Right) Body weight of the mice in various groups during the treatment. The data are presented as the mean ± SD (n = 4).

**Table S1.** $^{1}H$ NMR spectroscopic data (δ) for 3-butylaminophthalonitrile, 4-butylaminophthalonitrile, ***α*-Pc** and ***β*-Pc**.

| **Compound (Solvent)** | **δ** |
|---|---|
| 3-butylaminophthalonitrile ($CDCl_3$) | 7.47-7.43 (t, 1H,-$C_6H_3$-), 7.01-6.99 (d, 1H,-$C_6H_3$-), 6.91-6.89 (d, 1H, -$C_6H_3$-), 4.81 (s, 1H, -NH-), 3.25-3.21 (t, 2H, -$CH_2$-), 1.70-1.63 (m, 2H, -$CH_2$-), 1.48-1.43 (m, 2H, -$CH_2$-), 1.00-0.96 (t, 3H, -$CH_3$) |
| 4-butylaminophthalonitrile ($CDCl_3$) | 7.51-7.49 (d, 1H,-$C_6H_3$-), 6.83 (s, 1H,-$C_6H_3$-), 6.75-6.73 (d, 1H, -$C_6H_3$-), 5.34 (s, 1H, -NH-), 3.19-3.16 (t, 2H, -$CH_2$-), 1.68-1.60 (m, 2H, -$CH_2$-), 1.45-1.40 (m, 2H, -$CH_2$-), 1.00-0.96 (t, 3H, -$CH_3$). |
| ***α*-Pc** ($CDCl_3$/[$D_6$]DMSO (6:1)) | 7.80-6.58 (-$Pc_{C6H3}$-), 3.64-3.38 (4H, -NH-), 2.20-1.78 (-$CH_2$-), 1.18-0.79 (-$CH_2CH_2CH_3$), -1.95 (-$Pc_{NH}$-). |
| ***β*-Pc** ( [$D_6$]DMSO) | 8.95-8.82 (4H, -$Pc_{C6H3}$-), 8.37-8.20 (4H, -$Pc_{C6H3}$-), 7.38-7.34 (4H, -$Pc_{C6H3}$-), 6.96-6.79 (4H, -NH-), 3.44-3.30 (-NH$\underline{CH_2}$-), 1.81-0.93 (28H, -$CH_2CH_2CH_3$). |

**Table S2.** Summary of recent typical Pc-nanomaterials for PTT/PDT synergistic phototherapy in living cells by a single light source.

| sample | light source ($W{\cdot}cm^{-2}$) | η (%) | PS ($\mu$M) | MTT (%) | ref. |
|---|---|---|---|---|---|
| Pc@HSNs | 730 nm (1.5) | 37 | ~575 | 30 | 1 |
| NanoPcTB | 655 nm (2.5) | -- | 6 | 20 | 2 |
| ZnPc NW | 808 nm (3.0) | -- | ~205 | 50 | 3 |
| $O_2$@PFH@HMo$S_x$-HSA/AlPc | 670 nm (1.0) | 40 | ~255 | 25 | 4 |
| GR-TSCuPc | 650 nm (3.0) | -- | ~15 | 40 | 5 |

| | | | | | |
|---|---|---|---|---|---|
| SWNHs−TSCuPc | 650 nm (3.0) | -- | ~15 | 15 | 6 |
| ZnPc NPs | 650 nm (0.7) | 31 | 20 | ~15 | 7 |
| **HSA-*β*-Pc** | 808 nm (1.5) | 54 | 20 | 31 | this work |
| **HSA-*α*-Pc** | 808 nm (1.5) | 56 | 20 | 20 | this work |

**References**


1. J. Peng, L. Zhao, X. Zhu, Y. Sun, W. Feng, Y. Gao, L. Wang and F. Li, *Biomaterials*, 2013, **34**, 7905-7912.
2. X. Li, C. Y. Kim, S. Lee, D. Lee, H. M. Chung, G. Kim, S. H. Heo, C. Kim, K. S. Hong and J. Yoon, *J. Am. Chem. Soc.*, 2017, **139**, 10880-10886.
3. H. K. Moon, M. Son, J. E. Park, S. M. Yoon, S. H. Lee and H. C. Choi, *NPG Asia Materials*, 2012, **4**, e12-e12.
4. J. Wang, L. Liu, Q. You, Y. Song, Q. Sun, Y. Wang, Y. Cheng, F. Tan and N. Li, *Theranostics*, 2018, **8**, 955-971.
5. B.-P. Jiang, L.-F. Hu, D.-J. Wang, S.-C. Ji, X.-C. Shen and H. Liang, *J. Mater. Chem. B*, 2014, **2**, 7141-7148.
6. B. P. Jiang, L. F. Hu, X. C. Shen, S. C. Ji, Z. Shi, C. J. Liu, L. Zhang and H. Liang, *ACS Appl. Mater. Inter.*, 2014, **6**, 18008-18017.
7. Z. Wang, S. Gai, C. Wang, G. Yang, C. Zhong, Y. Dai, F. He, D. Yang and P. Yang, *Chem. Eng. J.*, 2019, **361**, 117-128.
